# The Rhinobiome of Exacerbated Wheezers and Asthmatics: Insights From a German Pediatric Exacerbation Network

**DOI:** 10.3389/falgy.2021.667562

**Published:** 2021-05-31

**Authors:** Malik Aydin, Cornelius Weisser, Olivier Rué, Mahendra Mariadassou, Sandra Maaß, Ann-Kathrin Behrendt, Yan Jaszczyszyn, Tatje Heilker, Maximilian Spaeth, Silvia Vogel, Sören Lutz, Parviz Ahmad-Nejad, Viktoria Graf, Aliyah Bellm, Christoph Weisser, Ella A. Naumova, Wolfgang H. Arnold, Anja Ehrhardt, Almut Meyer-Bahlburg, Dörte Becher, Jan Postberg, Beniam Ghebremedhin, Stefan Wirth

**Affiliations:** ^1^Laboratory of Experimental Pediatric Pneumology and Allergology, Faculty of Health, Center for Biomedical Education and Research, School of Life Sciences, Witten/Herdecke University, Witten, Germany; ^2^Center for Child and Adolescent Medicine, Helios University Hospital Wuppertal, Witten/Herdecke University, Wuppertal, Germany; ^3^Université Paris-Saclay, INRAE, MaIAGE, Jouy-en-Josas, France; ^4^Center of Functional Genomics of Microbes, Institute of Microbiology, University of Greifswald, Greifswald, Germany; ^5^Pediatric Rheumatology and Immunology, Department of Pediatrics, University Medicine Greifswald, Greifswald, Germany; ^6^Université Paris-Saclay, CEA, CNRS, Institute for Integrative Biology of the Cell, Gif-sur-Yvette, France; ^7^Department of Pathology, Division of Molecular Pathology, Helios University Hospital Wuppertal, Center for Clinical and Translational Research, Witten/Herdecke University, Wuppertal, Germany; ^8^Children's Hospital, Helios Hospital Niederberg, Teaching Hospital of University Hospital Essen, Velbert, Germany; ^9^Institute for Medical Laboratory Diagnostics, Center for Clinical and Translational Research, Helios University Hospital Wuppertal, Witten/Herdecke University, Wuppertal, Germany; ^10^Helios Hospital Krefeld, Children's Hospital, Teaching Hospital of Rheinisch-Westfälische Technische Hochschule (RTWH) University Aachen, Krefeld, Germany; ^11^Department of Economics and Center for Statistics, Georg-August University Göttingen, Göttingen, Germany; ^12^Department of Biological and Material Sciences in Dentistry, Faculty of Health, Witten/Herdecke University, Witten, Germany; ^13^Department of Human Medicine, Faculty of Health, Institute of Virology and Microbiology, Center for Biomedical Education and Research, Witten/Herdecke University, Witten, Germany; ^14^Laboratory of Clinical Molecular Genetics and Epigenetics, Faculty of Health, Center for Biomedical Education and Research, School of Life Sciences, Witten/Herdecke University, Wuppertal, Germany

**Keywords:** nasal microbiome, proteomics, metagenomics, bioinformatics, bacteria, *Moraxella catarrhalis*, asthma, exacerbation

## Abstract

Although the nose, as a gateway for organism–environment interactions, may have a key role in asthmatic exacerbation, the rhinobiome of exacerbated children with asthma was widely neglected to date. The aim of this study is to understand the microbiome, the microbial immunology, and the proteome of exacerbated children and adolescents with wheeze and asthma. Considering that a certain proportion of wheezers may show a progression to asthma, the comparison of both groups provides important information regarding clinical and phenotype stratification. Thus, deep nasopharyngeal swab specimens, nasal epithelial spheroid (NAEsp) cultures, and blood samples of acute exacerbated wheezers (WH), asthmatics (AB), and healthy controls (HC) were used for culture (*n* = 146), 16 S-rRNA gene amplicon sequencing (*n* = 64), and proteomic and cytokine analyses. Interestingly, *Proteobacteria* were over-represented in WH, whereas *Firmicutes* and *Bacteroidetes* were associated with AB. In contrast, *Actinobacteria* commonly colonized HCs. Moreover, *Staphylococcaceae, Enterobacteriaceae, Burkholderiaceae, Xanthobacteraceae*, and *Sphingomonadaceae* were significantly more abundant in AB compared to WH and HC. The α-diversity analyses demonstrated an increase of bacterial abundance levels in atopic AB and a decrease in WH samples. Microbiome profiles of atopic WH differed significantly from atopic AB, whereby atopic samples of WH were more homogeneous than those of non-atopic subjects. The NAEsp bacterial exposure experiments provided a disrupted epithelial cell integrity, a cytokine release, and cohort-specific proteomic differences especially for *Moraxella catarrhalis* cultures. This comprehensive dataset contributes to a deeper insight into the poorly understood plasticity of the nasal microbiota, and, in particular, may enforce our understanding in the pathogenesis of asthma exacerbation in childhood.

## Introduction

Childhood asthma is one of the most common respiratory disease worldwide, which is characterized by airway inflammation and reversible respiratory symptoms (1, 2). Asthma and wheeze during childhood are complex disease entities; their clinical presentation is shadowed by multifactorial causes (3). Increased westernized lifestyle conditions in urban environment, air pollution, and the method of childbirth may contribute to this dramatic prevalence (4–7). Infections with certain bacteria in early childhood may at least indirectly be a risk factor for asthma, and the inclusion of microbiome phenotypes as biomarkers may eventually contribute to an improved understanding of disease pathogenesis, course monitoring, comparisons, and similarities between wheezers (WH) and asthmatics (AB) (8, 9). In detail, the microbiome of the nasopharynx (NP) may develop from bacterial species that are colonized on the skin or vaginal flora of mothers and may be transmitted from parents to their children (10–13). During the first year of life, the bacterial colonization changes through contact with other individuals due to the use of antibiotic therapies; further bacterial species may be added, or certain bacterial species may be selected out of the microbiome (13–15). Interestingly, children with a neonatal colonization of *S. pneumoniae, M. catarrhalis*, and *H. influenzae* or a combination of these bacteria are at increased risk of developing persistent wheeze or asthma (16). The microbiome of the NP and the oropharynx (OP) is similar to that of the lung (9, 17, 18). Children with asthma have a lower bacterial diversity in the NP compared to healthy subjects (19). Although few studies describe the role of nasal and pharyngeal microbiome in patients with allergies and asthma and indicate that the microbiome may have an influence on the regulatory units of the innate immune system (20, 21), the characterization of the nasal microbiome in exacerbated children with chronic bronchitis and asthma is not sufficiently well-studied. Moreover, the literature does not embrace the early childhood exacerbation, which should reflect the burden of asthma, as stated by the World Health Organization (https://www.who.int/news-room/fact-sheets/detail/asthma). A better in-depth knowledge of the factors triggering acute exacerbations are important. Hence, considering these aspects, we aimed to describe the bacterial composition of the nasal cavity of exacerbated pediatric WH and AB and analyzed the impact of *M. catarrhalis* on the proteome profile of nasal epithelial spheroids (NAEsp) with the main desire to improve the clinical stratification in the near future.

## Materials and Methods

### Subjects and Study Design

We performed a prospective clinical trial of acute exacerbated pediatric subjects with chronic bronchitis/wheeze (3 months to <6 years of age) or asthma (≥6–17 years of age) suffering from an acute exacerbation episode. A detailed study description was previously published (22, 23). Briefly, deep nasopharyngeal swab specimens were taken during study enrollment. The AB and WH were divided into atopic and non-atopic groups based on clinical and laboratory parameters (e.g., positive allergic sensitization, total IgE levels, IgE-specific antibodies to distinct allergens, positive clinics for atopic disorders, etc.). In addition, children without any chronic diseases, absent from acute febrile infections, and term delivery were classified as healthy subjects (22, 23).

### Technical Information

#### 16S rRNA Meta-Barcoding and Culturing

To analyze the bacterial diversity in the nasopharyngeal cavity using the 16S V3–V4 ribosomal RNA regions, data from our pediatric exacerbation study cohort were used for nasal microbiota analyses through high-throughput next-generation sequencing (NGS) technique. Deep frozen swab specimens (eSwab COPAN LQ Amies) were systematically thawed. For DNA extraction, a special protocol was established using QIAmp DNA micro and QIAmp DNA Microbiome kits from QIAGEN. Further information on the DNA isolation method is available in this article's online repository material. For amplification of the V3 and V4 regions of the bacterial DNA, the 16S gene amplicon sequencing library preparation protocol from Illumina^®^ was used according to Illumina's recommendations. The primer sequences used for this study were already previously tested as most effective for 16S ribosomal RNA V3 and V4 regions (22, 24, 25) (https://emea.support.illumina.com/documents/documentation/chemistry_documentation/16s/16s/16s-metagenomic-library-prep-guide-15044223-b.pdf). A detailed technical information on library preparation is available in this article's online repository material.

The libraries were sequenced on an Illumina^®^ MiSeq instrument in a paired end 2 × 300 bp run, using a MiSeq Reagent Kit v3 (600 cycle). Demultiplexing was performed (bcl2fastq2 V2.2.18.12) and adapters were trimmed with Cutadapt 1.15; only reads longer than 10 bp were kept. For each sample, at least 75,000 reads were obtained.

For the classical bacterial culture, each nasopharyngeal swab was streaked on BD™ Chocolate Agar, Columbia blood with 5% sheep blood, Columbia CNA Agar, and MacConkey Agar (all media from Becton Dickinson, Heidelberg) for up to 48 h incubation at 37°C. The blood-containing medium was incubated at 37°C and 5% CO_2_ atmosphere (Panasonic, Inco Safe CO_2_ Incubator, MCO-230AICUV). Matrix-assisted laser desorption/ionization time-of-flight mass spectrometry (MALDI-TOF-MS, Bruker) was used for bacterial identification based on ribosomal proteins. The antimicrobial susceptibility testing of the bacterial isolates was performed either with the automated system BD Phoenix™ or manually via an Epsilometer test, and/or agar diffusion tests.

#### Moraxella Catarrhalis, Staphylococcus Aureus, and Haemophilus Influenzae *in vitro* Exposure Model

To analyze the effects of isolated *M. catarrhalis* on nasal epithelial cells (NAEPCs), subject-derived NAEPCs at passage 2 (P2) were seeded for spheroid cell culture (1 × 10^3^ cells per well) on a non-cell attachment, lipidure^®^-coated, 96-well, U-bottom plate from amsbio^®^ using Pneumacult ALI culture media from STEMCELL™ Technologies, Vancouver, Canada (26). The culture media was changed every 2 days, and the cells were incubated at 37°C and 5% CO_2_ for up to maximum 10 days until a size of ~200–300 nm was observed. Subject-derived deep-frozen *M. catarrhalis* cultures of AB and WH were thawed and re-cultivated on a Choco culture agar plate for 24 h at 37°C and 5% CO_2_. A biofilm production was confirmed using the 96-well microtiter plate assay. Following incubation at 37°C, planktonic bacteria were rinsed with sterile saline, and the remaining adherent bacteria were stained with crystal violet dye. Biofilm-producing *M. catarrhalis* cultures were used in a concentration of 5 × 10^5^ per well on spheroid cell cultures and incubated for 24 h at 37°C and 5% CO_2_. In addition, *S. aureus* and *H*. (*para-)influenzae* cultures were also used for comparison experiments. Epithelial cell damage factors such as lactate dehydrogenase (LDH) were assessed for *M. catarrhalis* exposure experiments using LDH-Glo™ cytotoxicity assay provided by Promega. Using bead-based multiplex LEGENDplex™ assays by BioLegend, Canada (Human B cell Panel #740527, Human Cytokine Panel 2 #740526, Human Inflammation Panel 1 #740808), the following cytokines were analyzed: Interleukin-(IL)17A, IFN-γ, and TNF-α. LEGENDplex™ assay kits were performed according to the manufacturer's instructions. In detail, samples were incubated with capture beads and assay buffer in V-bottom plates overnight with constant shaking at 4°C. In parallel, standard dilutions were prepared and likewise incubated with beads and Matrix A1. After two washing steps (800 × g for 5 min with 200 μl of wash buffer), beads were incubated with biotinylated detection antibodies for 1 h followed by streptavidin-PE for 30 min at RT on a plate shaker. Plates were washed and beads were resuspended in 100 μl of wash buffer. Samples were stored protected from light at 4°C until measurement with a BD FACS Canto II flow cytometer. Reactions were performed in duplicate. Data were analyzed via LEGENDplex™ V8.0 software (BioLegend^®^) based on standard curve interpolation and specified as pg/ml.

#### Whole Proteome Analyses

Next, we were interested whether the proteome level of infected NAEsp with subject-derived *M. catarrhalis* was biologically different between groups. The exposure experiments with *M. catarrhalis* were firstly lysed, and the extracted proteins were separated by 1D SDS-PAGE before tryptic in-gel digestion. Eluted peptides were analyzed by LC-MS/MS using an EASY-nLC II liquid chromatography system coupled to an LTQ Orbitrap Velos Pro (ThermoFisher Scientific, Waltham, Massachusetts, U.S.A.). Results obtained during analysis of a pure culture of *n* = 5 subject-derived *M. catarrhalis* strains were used to create a spectral library, which was subsequently used to identify spectra obtained from infection experiments. First, the data obtained from infection experiments were searched against the generated spectral library using the tool “SpectraST Search” within the TPP framework. Obtained pep.xml files where combined to interact.pep.xmls and processed with Peptide Prophet. Resulting ipro.pep.xmls were merged into protein lists using the TPP Tool “Analyze proteins” with activated “Normalize NSP using protein length” option. Finally, the software Philosopher (Nesvizhskii Lab) was used to filter the prot.xml files for an FDR of <0.01 on peptide and protein level. More details on sample preparation, MS analysis, and data processing can be found in the Supplementary Material.

### Bioinformatics Analyses

Statistical analyses for LDH and cytokine measurements were performed using GraphPad Prism version 8.3.0 for Windows, GraphPad Software, La Jolla, California, U.S.A. (www.graphpad.com). The data were presented as mean and standard error of the mean (SEM). The data were firstly tested for normal distribution using the Shapiro–Wilk test. The majority of the results were not normally distributed. To perform statements regarding significant differences between groups, the following non-parametric test procedures were therefore used: for >2 groups, the Kruskal–Wallis test with Dunn's multiple *post-hoc* test was used for correction for multiple comparisons; for simple pair comparisons (two groups), the Mann–Whitney *U*-test was used. The significance level α was set as 0.05. The significance level was set at ^*^p < 0.05.

The quality of the sequenced samples was assessed with FASTQC Babraham Bioinformatics (http://www.bioinformatics.babraham.ac.uk/projects/fastqc) (27). Sequencing data analyses were performed with FROGS pipeline (27). FROGS is designed to analyze large sets of amplicon sequences and produces abundance tables of operational taxonomic unit (OTU) and their taxonomic affiliation. Briefly, this pipeline includes a pre-processing step where reads were merged with PEAR (28), primers were removed with cutadept, and remaining sequences were de-replicated and filtered according to their length (400–440 bp) and N content. This step was followed by Swarm clustering (29) with an agglomeration distance of *d* = 3. Chimera detection was then performed using Vsearch (30) before applying an OTU abundance filter (OTUs with total count <10 × e5 of the total abundance were discarded). The sequence of each OTU was then affiliated with blastn against the Silva v132 database (31). OTUs with a blast coverage <95% were removed. Finally, Fasttree (32) was used to build a phylogenetic tree from the remaining sequences.

All diversity analyses were performed using the R statistical software (v 3.6.1) and phyloseq package (https://www.R-project.org/) (33). α-diversity was computed using the specific richness, the Shannon index (moderately upweights abundant taxa), and the Inverse-Simpson (upweights abundant taxa) index. α-diversity indices were compared with Kruskal–Wallis tests, followed by Dunn's *post-hoc* tests. β-diversity was computed using the Jaccard, Bray–Curtis (upweights abundant taxa), UniFrac (upweights phylogenetically distant taxa), and weighted UniFrac (upweights phylogenetically distant and abundant taxa) indices after rarefaction to the same number of reads per sample. Ordination of β-diversity matrices to produce 2D-plots was done using multidimensional scaling (MDS), and PERMANOVA (a multivariate equivalent of ANOVA) was used to test whether a factor had an effect on the average microbiome composition.

Furthermore, DESeq2 was used to perform the differential abundance tests. DESeq2 uses a generalized linear model with negative binomial distribution. The *p-*values were corrected for multiple comparison using the Benjamini–Hochberg correction procedure to control the false discovery rate at a *p*-level of 0.05. The final normalized read number was 50,000 reads per sample.

## Results

### The Characterization of the Patient Cohort

For this approach, *n* = 146 nasopharyngeal swab specimens were used for further analyses including culture-based (*n* = 146) and NGS-based methodology (*n* = 64). A total of 146 patient samples were used for cultural analyses, whereas *n* = 46 was derived from AB (*n* = 17♀; 10.3 ± 3.3 years), *n* = 61 from WH (*n* = 20♀; 2.3 ± 1.5 years), and *n* = 39 from HC cohorts (*n* = 18♀; 8.5 ± 4.4 years). A total of 64 patient samples were additionally used for 16S-rRNA gene amplicon sequencing analyses, whereas *n* = 23 was derived from AB (*n* = 6 ♀, 9.4 ± 3.5 years), *n* = 23 from WH (*n* = 9 ♀, 2.6 ± 1.3 years), and *n* = 18 from HC groups (*n* = 9♀, 8.1 ± 3.4 years). Table 1 summarizes the patient-relevant characteristics in detail.

**Table 1:** Clinical item parameters of samples used for this approach.

	**Asthmatics**	**Wheezers**	**Healthy controls**
	***n* = 46**	***n* = 61**	***n* = 39**
Atopic status positive (yes, %)	41 (89.1%)	25 (41.0%)	0 (0.0%)
Chronic steroid intake (yes, %)	18 (39.1%)	18 (29.5%)	0 (0.0%)
Environmental risk factors i.e., diesel exposure (yes, %)	15 (42.9.2%)	13 (23.6%)	5 (13.9%)
	*n* = 35	*n* = 54	*n* = 36
Tobacco/alcohol exposure during pregnancy (yes, %)	8 (22.2%)	13 (23.6%)	2 (5.6%)
	*n* = 36	*n* = 55	*n* = 36
Breast feeding (yes, %)	26 (74.3%)	41 (74.6%)	33 (91.7%)
	*n* = 35	*n* = 55	*n* = 36
Pets (yes, %)	9 (25.7%)	12 (22.2%)	10 (27.8%)
	*n* = 35	*n* = 54	*n* = 36
Mold (yes, %)	17 (50.0%)	15 (28.3%)	7 (19.4%)
	*n* = 34	*n* = 53	*n* = 36

*Values are presented as mean (M). Some item parameters were not fulfilled by subjects/custodians and are missing; each n-number is described below the percentages*.

### The Nasal Cavity of WH Is Colonized With More Bacteria Than Other Groups

By use of culture-based methodology, *n* = 146 nasal swab specimens were taken into the analyses. In detail, 46 of 61 subjects with wheeze had a positive bacterial culture (75.4%) in comparison to AB (*n* = 21 of 46) and HC (*n* = 12 of 39). Specifically, we detected *H. (para-)influenzae, Moraxella* spp., *Streptococcus* spp., and *Staphylococcus* spp. in the specimens of our cohort. In concordance with the literature, WH showed more colonization with *M. catarrhalis* [*n* = 21 (45.6%) vs. *n* = 4 (19.1%) in AB, and *n* = 3 (25%) in HC], as well as *H. (para-)influenzae* [*n* = 20 (43.5%) vs. *n* = 8 (38.1%) in AB, and *n* = 3 (25%) in HC], *S. pneumoniae* [*n* = 3 (6.5%) vs. *n* = 0 (0%) in AB, and *n* = 0 (0%) in HC], and *S. aureus* [*n* = 11 (23.9%) vs. *n* = 11 (52.4%) in AB, and *n* = 6 (50%) in HC]. The culture-based results were again divided into further subgroups (atopic/non-atopic) and analyzed. Interestingly, atopic AB (*n* = 41) showed positive cultures for *H. (para-)influenzae* in *n* = 7 cases, *n* = 10 for *S. aureus*, and *n* = 2 for *M. catarrhalis*, whereas atopic WH (*n* = 25) showed similar results: *n* = 8 positive cultures for *H. (para-)influenzae, n* = 8 for *S. aureus*, and *n* = 12 for *M. catarrhalis*. When the respective groups were divided into non-atopic subgroups, non-atopic AB (*n* = 5) showed *n* = 1 positive cultures for *H. (para-)influenzae, n* = 2 for *S. aureus*, and *n* = 1 for *M. catarrhalis*. The non-atopic WH (*n* = 36) had *n* = 12 positive cultures for *H. (para-)influenzae, n* = 3 for *S. aureus*, and *n* = 9 for *M. catarrhalis*.

### The Composition of the Nasal Microbiota Varied Between WH and AB

**Figure 1:**
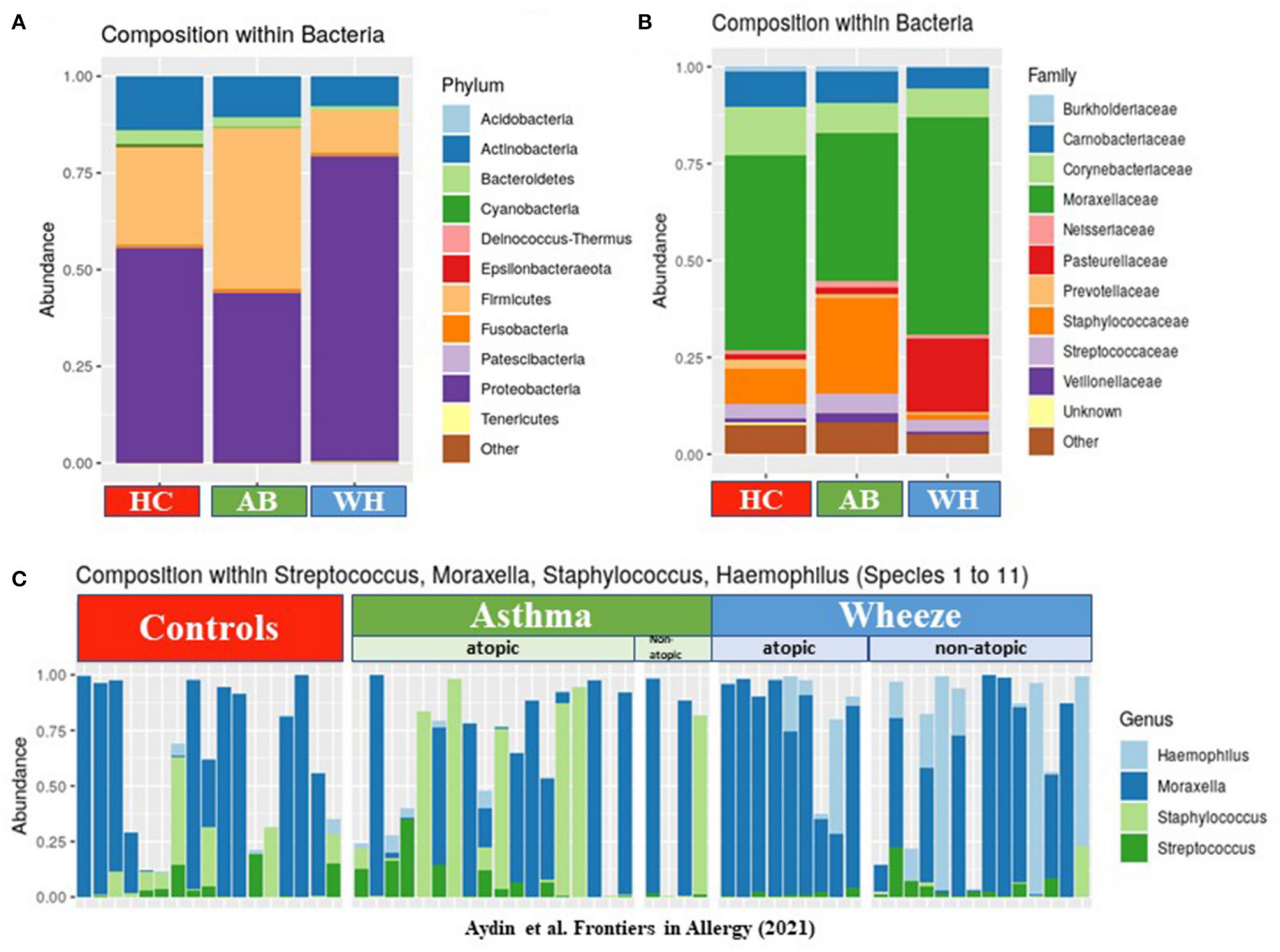
The composition of bacteria in different disease groups. **(A)** The *y*-axis shows the abundances of each phylum level. *Proteobacteria* were highly abundant in WH, whereas *Firmicutes, Cyanobacteria*, and *Actinobacteria* were commonly more colonized in AB (*n* = 64). **(B)** This figure presents the genera level abundances of bacteria. **(C)** The relative abundance levels of *Moraxella* (and other genus found by cultural approach) are presented in three different groups: HC, AB, and WH and subclassified in atopic/non-atopic AB and WH (AB, Asthmatics; WH, Wheezers; HC, Healthy controls).

Next, nasopharyngeal swab specimens were then used for 16S-rRNA gene amplicon sequencing analyses. Here, the abundance of phyla revealed differences between AB, WH, and HC (Figure 1A). In detail, *Proteobacteria* was more frequently sequenced in WH. Asthmatics, on the other hand, had more *Firmicutes*. *Actinobacteria* was more frequently present in the HC group (permANOVA *p* = 0.005). Figure 1B presents the genera level abundances of bacteria. Categorizing the subjects into atopic and non-atopic status, atopic WH had more commonly *Proteobacteria* in the nasal swab specimens than atopic AB (permANOVA *p* = 0.015). *Moraxella* and *Haemophilus* were among the most abundant genera in WH. In detail, *S. pneumoniae, M. catarrhalis, S. aureus*, and *Haemophilus (para-) influenzae* accounted for most of the sequences. Interestingly, further analyses of *Proteobacteria* revealed that non-atopic WH were more frequently colonized with *Moraxella* and *Haemophilus* as compared to AB and HC. Atopic AB were more colonized with *Streptococcus* and *Staphylococcus* in the nasal cavity. Atopic WH had higher *Moraxella* colonization rates than AB, whereas non-atopic WH were more colonized with *Streptococcus* and *Moraxella* colonization (Figure 1C). We then compared the colonization levels of several taxa between atopic subjects with HC. *Staphylococcaceae* (*p* < 0.05), *Enterobacteriaceae* (*p* < 0.05), *Burkholderiaceae* (*p* < 0.05), *Xanthobacteriaceae* (*p* < 0.05), and *Sphingomonadaceae* (*p* < 0.05) were significantly more abundant in atopic pediatric subjects (Supplementary Figures 1A,B).

### α-Diversity Level Differed in Terms of Atopic Level

**Figure 2:**
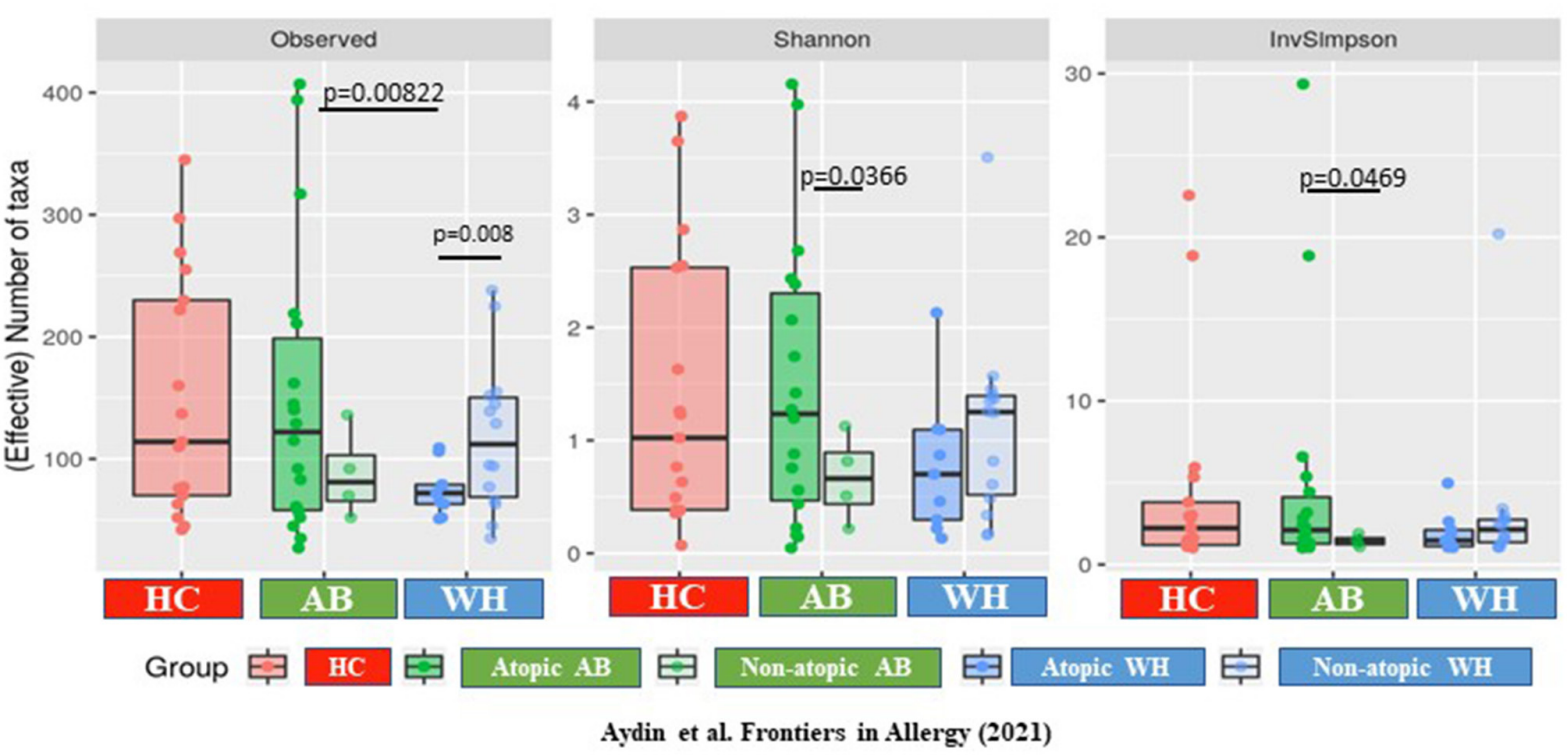
The α-diversity differs between atopic and non-atopic diseased subjects. The α-diversity is increased in atopic AB but is decreased in WH (Observed *p* = 0.008, Shannon *p* = 0.066, InvSimpson *p* = 0.081) (AB, Asthmatics; WH, Wheezers; HC, Healthy controls).

Focusing on the specific α-diversity using the observed number of OTUs, according to the Shannon (moderately upweights abundant taxa) and the Inverse-Simpson indices (strongly upweights abundant taxa), observed on the rarefaction curves, the diversities were widely distributed in terms of number of OTU, but less so in terms of number of effective taxa (InvSimpson). The atopic WH were different from the atopic AB, as the atopic from the non-atopic WH (*p* = 0.0198) and the atopic from the non-atopic AB (*p* = 0.0198; Supplementary Figures 2A,B). Thus, the α-diversity was increased in atopic AB, but it was decreased in WH (Observed *p* = 0.008, Shannon *p* = 0.066, InvSimpson *p* = 0.081) (Figure 2). The β-diversity results are presented in the Supplementary Material of this manuscript.

### Differential Abundances of Taxa Showed Distinct Clusters in the AB, WH, and HC

We filtered the taxa (keeping only those present in at least 12% of the samples) before performing a differential abundance analysis on the remaining taxa. In total, we identified *n* = 24 OTUs with differential abundances in AB, WH, and HC (Supplementary Figures 3A,B and Supplementary Table 1). With reference to the atopic status, we observed *n* = 42 OTUs with significant differential abundances in atopic AB and WH (Supplementary Figures 3C,D and Supplementary Table 2). The filter applied to the differential abundance analysis and the rationale of the filter was to increase statistical power by pruning the rare taxa whose prevalence was too low in the dataset for any test to be significant. Thus, the 12% prevalence threshold correspond to a taxa being present in at least eight samples. First and foremost, below that prevalence threshold, abundance data are scarce and the statistical power to detect meaningful differences between groups is small, so that discoveries made on those taxa will disproportionately be false positives. Additionally, and since we correct for multiple testing issues, including those taxa in the differential analyses increases the total number of tests performed and thus decreases the statistical power for more prevalent taxa. Finally, taxa with very low prevalence are unlikely to correspond to biologically relevant effects. This method as filtering prior to differential analysis is quite common in the field to increase the statistical power.

### Moraxella Catarrhalis may Be Responsible for Epithelial Damage in Human Primary NAEPC Culture

**Figure 3:**
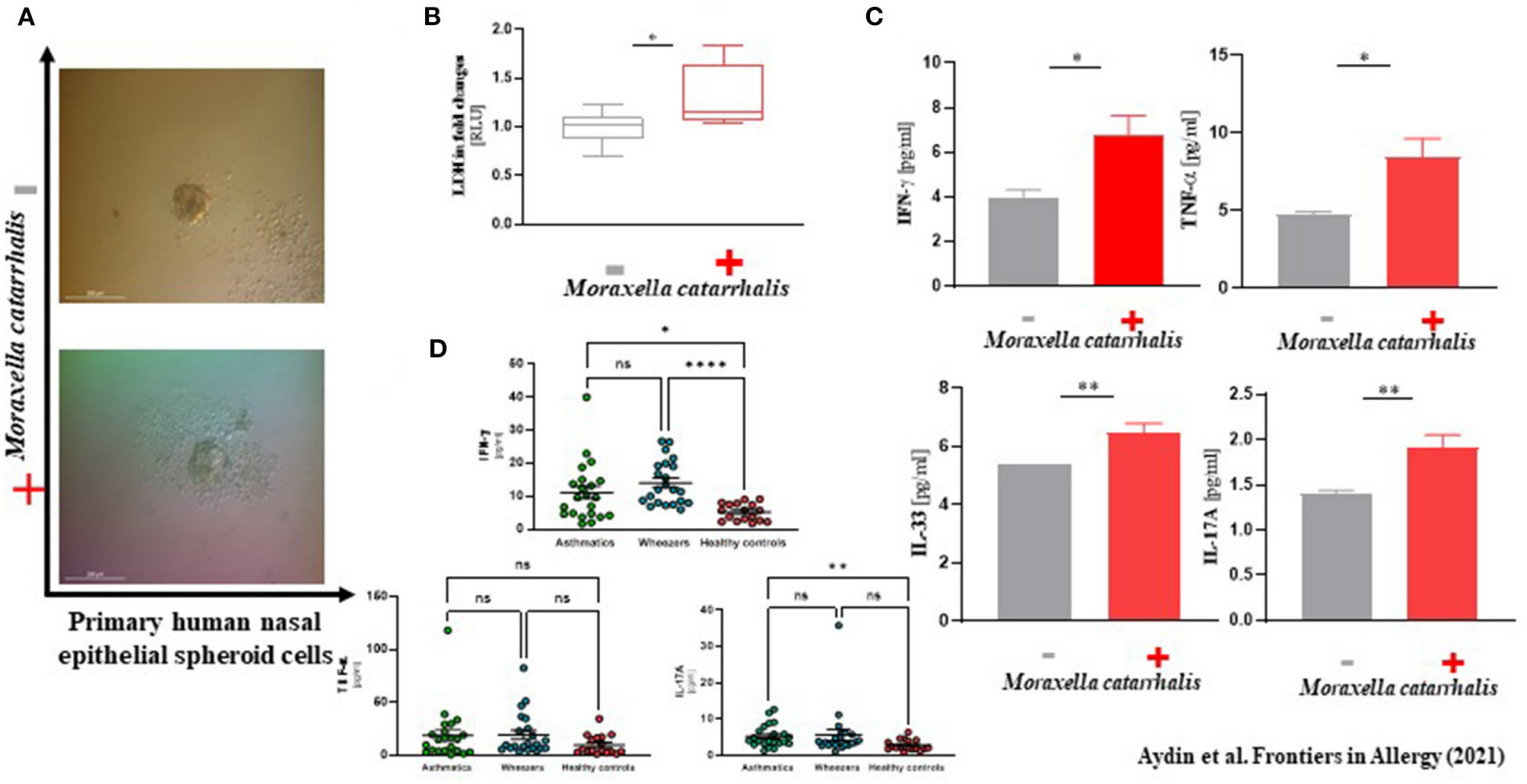
Phenotype-specific *Moraxella catarrhalis* infection could be associated with a disruption of epithelial integrity *in vitro*. **(A)** Primary human nasal epithelial cells were used for spheroid (NAEsp) cell culture technique (1 × 10^3^ cells per well). After circa 1 week, NAEsp reached a size of 200–300 nm and were used for infection experiments with *M. catarrhalis* strains derived from AB and WH in a concentration of 5 × 10^5^ cfu per NAEsp. After 24 h of infection, the epithelial barrier of the spheroids was microscopically impaired. **(B)** Lactate dehydrogenase (LDH), as a potential epithelial damage factor, was increased in supernatants of provoked and non-provoked NAEsp with *M. catarrhalis* (normalized values presented as fold changes). **(C)** The concentration of IL-33, IFN-γ, TNF-α, and IL-17A was significantly elevated in supernatants of the exposure group with *M. catarrhalis* (*the non-provoked cells showed values under the detection and the measurable reference values were used for analyses*). **(D)** Furthermore, we then analyzed IFN-γ, TNF-α, and IL-17A in plasma samples of the study population using LEGENDplex™ bead assay. Concordantly, our pediatric exacerbation study cohort revealed a significant difference between exacerbated AB, WH, and HC, specifically for IFN-γ and IL-17A (AB, Asthmatics, *n* = 22; WH, Wheezers, *n* = 22; HC, Healthy controls, *n* = 17). ^*^p < 0.05, ^**^p < 0.01, ^***^p < 0.001, ^****^p < 0.0001.

In our analyses, *M. catarrhalis* were commonly more present in the nasal cavity of WH compared to AB and HC. Considering the literature of the potential clinical relevance of *M. catarrhalis*, we aimed to analyze its influence on the epithelial integrity and proteome level by performing an *in vitro* exposure experiment with subject-derived *M. catarrhalis* strains on NAEsp. Here, we investigated histological, cytokine, and proteome analyses after exposure. We infected NAEsp from HC donors with isolated subject-derived *M. catarrhalis* strains, which were previously cultured from AB and WH groups. Interestingly, 24 h post-infection, we observed a disruption of the epithelial integrity (Figure 3A). Moreover, epithelial damage factors (i.e., lactate dehydrogenase) and the concentration of IFN-γ, TNF-α, and IL-17A were significantly released in *M. catarrhalis* exposure experiments compared to wild-type (untreated, UT) NAEsp (Figures 3B,C). Furthermore, we performed cytokine analyses in plasma samples of the study cohort. Interestingly, the cytokine measurement provided significant differences between exacerbated AB and WH in comparison to healthy pediatric donors (Figure 3D). Additional exposure experiments with *H. influenzae* and *S. aureus* also showed an affection of the epithelial barrier for both bacterial strains, and a cytokine release in supernatants of *S. aureus*-related exposure experiments. *H. influenzae* was not significantly associated with cytokine release, although the infection was macroscopically observed by turbidity of the culture medium the day after infection and the cell integrity was also affected as observed in the *M. catarrhalis* and *S. aureus* exposure experiments (Supplementary Figure 4).

### tRNA-cytidine-2-sulfurtransferase may Be an Interesting Pathogenesis Marker for WH With Acute Exacerbation

**Figure 4:**
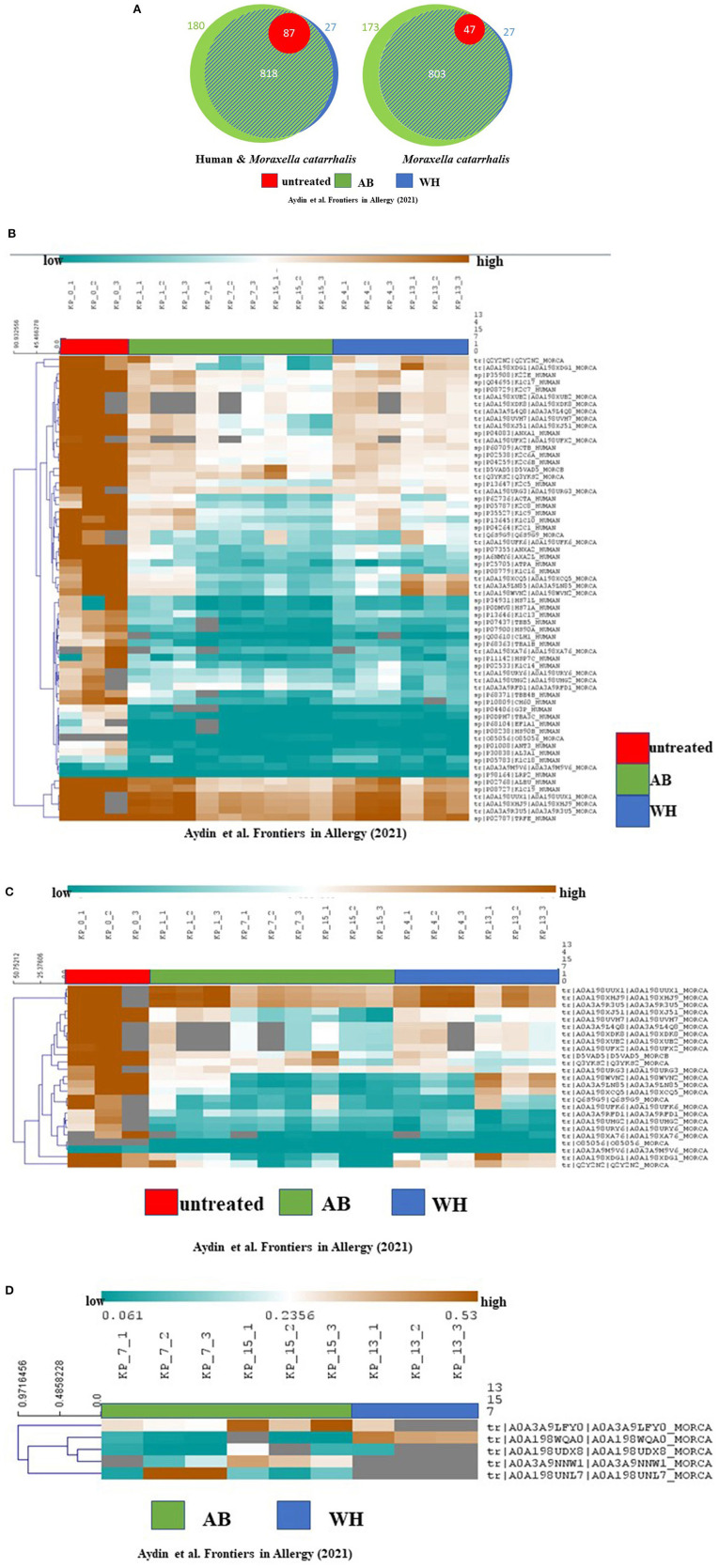
Proteome analyses revealed cohort-specific differences and TtcA was associated with WH. **(A)** Proteome analyses of the cultures showed the following quantified proteins (left: human and *M. catarrhalis*; right: *M. catarrhalis*) highlighted in a Venn diagram, depicted in unteated (UT) (red), AB (green) and WH (blue). The size of the circular area is proportional to the number of quantified proteins in each group. Healthy controls had less protein differences compared to WH and AB. A distinct proportion in the AB group had cohort-specific proteome abundance levels. **(B)** Proteome analyses of the cultures including human and *M. catarrhalis*-associated proteins presented significant abundance levels in AB, WH, and UT groups. The abundance levels are colored ranging from blue (low abundance) to orange (high abundance). Gray color represents missing information for each protein in the group. Interestingly, AB, WH, and UT had common high abundance levels for ALBU_Human, K1C19_Human, A0A198UUX1_MORCA, A0A198XHJ9_MORCA, A0A349RU5_MORC, and TRFE_Human. WH-specific high abundance levels were furthermore observed for different *M. catarrhalis* specific protein levels and this level was found to be reduced in AB, except for D5VAD5_MORCA and Q3YKS2_MORCA. **(C)** Spearman rank correlation was performed for clustering samples. A separate analysis for solely *M. catarrhalis*-specific proteins revealed significant correlations between AB, WH, and UT, and *in toto n* = 25 proteins did cluster with each group. The abundance levels range from blue (low abundance) to orange (high abundance). In detail, AB and WH had different abundances in few protein levels. **(D)** A differentiated analysis for the WH group presented a group-specific abundance protein level in comparison to the AB group; in particular, tRNA-cytidine-2-sulfurtransferase (TtcA), tr|A0A198WQA0|A0A198WQA0_MORCA, was highly significantly abundant only in the WH group (AB, Asthmatics; WH, Wheezers; HC, Healthy controls).

Next, to describe the role of *M. catarrhalis* during exacerbation in AB and WH, we were then interested to analyze the proteome profile of *M. catarrhalis* strains derived from the entire cohort. Subsequently, proteome analyses of the spheroid cell cultures infected with *M. catarrhalis* revealed discrete differences in the proteome profiles, particularly for samples of AB and WH groups compared to non-infected spheroid cell cultures. In detail, we observed disease-relevant proteome changes in *M. catarrhalis* exposure experiments (including human and *M. catarrhalis*-specific). The UT group had high abundance levels for *n* = 64 proteins except for the proteins K1C18_Human, A0A39M9V6_MORCAm, and LRP2_Human. Interestingly, AB, WH, and UT group had more common abundance levels for ALBU_Human, K1C19_Human, A0A198UUX1_MORCA, A0A198XHJ9_MORCA, A0A349RU5_MORC, and TRFE_Human. Wheezer-specific high abundance levels were furthermore observed for different *M. catarrhalis* specific protein levels, and these levels were reduced for AB, except for D5VAD5_MORCA and Q3YKS2_MORCA. The UT group had high protein abundance levels except for A0A3A9M9V6_MORCA and O85056_MORCA (Figures 4A,B). Asthmatics, WH, and UT group had high abundance levels for these three proteins, but in detail, AB and WH had different abundances in few proteins (Figure 4C). Strikingly, the detailed data inspection then showed that tRNA-cytidine-2-sulfurtransferase (TtcA), tr|A0A198WQA0|A0A198WQA0_MORCA was significantly abundant only in the WH compared to the AB group (Figure 4D).

## Discussion

The rhinobiome of children with exacerbated asthma and wheeze must be extensively studied. With this study, we aimed to characterize the nasal microbiota of children and adolescents with wheeze and asthma suffering from acute wheezing or asthmatic episode. In summary, we observed a variety of microbiome and proteome clusters, particularly in WH-derived samples.

The microbiome of the lung develops through micro-aspiration and inhalation of pathogens from the environment, the upper respiratory and the gastrointestinal tract, as well as through the reaction of the mucosa to these pathogens (15). It has already been shown that the microbiome is niche-specifically organized in complex communities and is strongly adapted to its environment (20). In our study population, *M. catarrhalis* as well as *S. aureus* and *H. influenzae* were predominantly colonized in the nasal cavity of WH. To determine whether these bacteria may be related to exacerbation, we analyzed the effects of these bacteria on healthy NAEsp cultures. Here, we observed cytokine release in cultures exposed to *S. aureus* and *M. catarrhalis*. Recently, McCauley and colleagues published an article on the microbiome of exacerbated pediatric asthmatics. They observed an increased colonization of *Moraxella, Haemophilus, Staphylococcus*, etc. in their cohort. To correlate their descriptive data, they analyzed the functionality of bacterial strains and observed increased cytokine levels in A549 lung cancer cells (34). In detail, *M. catarrhalis* significantly caused more epithelial damage and the greater release of cytokines when compared to other bacterial strains (34). Deducing ideas from these descriptive approaches, we then used *M. catarrhalis* strains to investigate proteome analyses. In detail, the proteome level of the exposure experiment with *M. catarrhalis* showed a difference between protein expression profiles in diseased groups. We observed that the whole proteome was phenotype-specific and that WH provided a somewhat different proteome composition during exacerbation *in vitro* compared to AB. In detail, TtcA, as a regulator of the post-transcriptional thiolation of the cytosine 32 in tRNA (35), was significantly abundant in WH-derived *M. catarrhalis* cultures in comparison to samples of exacerbated AB and HC groups. Moreover, the sulfate adenyltransferase subunit 1 and D-3-phosphoglycerate dehydrogenase were also abundant in our entire cohort. These proteins were specific to *M. catarrhalis*, and which influences on the human signaling pathway regarding asthma pathogenesis they have, are not fully understood. Thus, further analyses will soon give further insights into the relevance of these proteins and whether they have an influence on the pathogenesis of asthma exacerbation.

Mansbach et al. (36) have investigated the role of airway microbiota in their American multicenter study and analyzed the samples of infants after bronchiolitis over several time points. They observed that patients with bronchiolitis colonized with *Moraxella* or *Streptococcus* species were at increased risk of developing frequent wheezing episodes (36). Dumas et al. (37) showed a similar observation where infants with severe bronchiolitis and a colonization with *Haemophilus* and *Moraxella* showed later recurrent wheezing episodes in this group.

Several studies revealed that the microbiome may directly influence the regulatory units of the innate immune system and thus influence the pathophysiology of asthma. Additionally, there may also be phenotypic and regional differences in adult AB. Fazlollahi et al. (24) highlighted that at least in adults, ethnicity-dependent nasal microbiome differences seem to exist. They analyzed the 16 S-ribosomal nasal microbiome of adults with exacerbated and non-exacerbated AB in New York, U.S.A., and the nasal mucosa of adult AB was colonized with *Bacteroidetes* and *Proteobacteria*. *Prevotella buccalis, Dialister invisus, Gardnerella vaginalis*, and *Alkanindiges hongkongensis* were relatively more abundant as compared to the current asthma activity level (24). In view of individual variability, the differences might arise from the characteristics of the patients, the region where they grew up, and the age (24).

Many efforts have been focused on analyzing the link between microbiome and disease pathogenesis. *S. aureus* is one of the most well-studied bacteria in which this link is sufficiently described, as ~30% of all people worldwide are colonized with *S. aureus* (38, 39). In a recent publication by Tsilochristou et al. (40), a *S. aureus* colonization was strikingly connected to food allergies as well as to atopic eczema. In our cohort, the WH group was also more frequently colonized with this bacterium, perhaps indicating an increased likelihood of exacerbation.

Rosas-Salazar et al. (41) suggested that 40% of children with respiratory syncytial virus (RSV) infection in the first years of life may develop a wheeze and asthma in later years of age. Children with RSV in whom Lactobacillus could be detected in the NP/OP developed significantly fewer wheezing symptoms later in life. Thus, the occurrence of Lactobacillus in RSV-infected patients may reduce the risk of later wheezing (41). A meta-analysis of cesarean section deliveries and the risk of asthma showed that children who were delivered by cesarean section have a 20% higher risk of developing asthma compared to spontaneous vaginal deliveries (7, 42).

In our study population, we observed that the colonization of *H. influenzae* and *M. catarrhalis* was increased mainly in the preschool WH cohort. None of our cohorts received a long-term antibiotic treatment. Therefore, we assume that there is no bias that may influence the colonization of the nasal cavity. However, the question arises whether the use of several antibiotics in the history has a significant impact on the colonization in the nasal cavity. This information was not collected during study enrollment and must be taken into consideration during the interpretation of the data. Moreover, the effect of viral infection on disease pathogenesis and the microbiome composition have been extensively analyzed in the literature (43, 44). Moreover, there is also an interaction between viral infection and the microbial composition in the airways and an infection with RSV may be correlated with the colonization of *H. influenzae* or *Streptococcus* species (15). It should be considered that multifactorial conditions can lead to an exacerbation. In our study analysis, we did not perform respiratory viral panel testing yet, so this information is missing for this manuscript. Another limitation is that children who grew up in different milieus and have already undergone many antibiotic therapies may have a different microbiome profile than children of the same age or older/younger children as described previously in some studies. The same living and housing conditions, previous therapies, contact with animals, birth modes, etc. must be considered when analyzing the microbiome of patients. A substratification into the above factors to achieve a 1 to 1 matching makes the possibility of practical implementation difficult. Considering this fact, a potential age-based bias is not fully excluded. Nevertheless, we believe that it was important to include the wheezer study group into the analyses, as a defined percentage of this heterogeneous group will show a progress to asthma. Thus, this side information is critically important during interpretation of this dataset, which is necessary for an assessment of biodiversity. Longitudinal analyses of patients of whether bacterial colonization changes or differs between two study visits (exacerbation and phase without clinical symptoms) should be analyzed.

In summary, our complex dataset adds important insights into the plasticity of the nasal microbiome during exacerbation of pediatric chronic bronchitis and AB. Despite few limitations, the power of our entire cohort is important. Further ongoing studies and the inclusion of follow-up samples over different time frames would be interesting to address the role of the nasal microbiome in the pathogenesis of wheeze and asthma in order to characterize their impact on disease development.

## Data Availability Statement

The datasets presented in this study can be found in online repositories. The names of the repository/repositories and accession number(s) can be found below. The mass spectrometry proteomics data have been deposited to the ProteomeXchange Consortium via the PRIDE (45) partner repository with the dataset identifier PXD024694. Project Name: The rhinobiome of exacerbated preschool wheezers and asthmatics: insights from a German pediatric exacerbation network, Project accession: PXD024694. Moreover, the metagenomics data set are uploaded and have the following project number: BioProject ID: PRJNA714100.

## Ethics Statement

Ethical approval and its later amendments were obtained by the Ethics Committees of the Witten/Herdecke University (158/2017) and (Ärztekammer Nordrhein, NR 2019312), Germany, and the study was separately registered at the German Clinical Trials Register (DRKS) (registration number DRKS00015738). Written informed consent to participate in this study was provided by the subjects or/and the participants' legal guardian. This article does not contain any studies with animals performed by any of the authors.

## Author Contributions

MA, CoW, SM, DB, JP, BG, and SW had full access to all of the data in the study and take responsibility for the integrity of the data and the accuracy of the data analysis. MA, CoW, JP, BG, and SW: concept and design. MA, CoW, OR, MM, YJ, SM, DB, A-KB, TH, MS, SL, JP, AM-B, BG, and SW: acquisition, analysis, or interpretation of data. MA, CoW, JP, BG, and SW: drafting of the manuscript. MA, CoW, ChW, OR, MM, SM, and DB: statistical analysis. MA and SW: obtained funding. SV: administrative, technical, or material support. MA, JP, BG, and SW: supervision. All authors: critical revision of the manuscript for important intellectual content.

## Conflict of Interest

The authors declare that the research was conducted in the absence of any commercial or financial relationships that could be construed as a potential conflict of interest.

## Supplementary Material

Supplementary Figure 1 **(A,B)** The data presents the genus level taxonomy.

Supplementary Figure 2 The phylogenetic diversity. **(A)** The phylogenetic diversity contrasting pair with reads in HC, AB, and WH. **(B)** The atopic WH were significantly different from the atopic AB (*p* = 0.05), as well as the atopic vs. the non-atopic WH (*p* = 0.0198), and the atopic vs. non-atopic AB (*p* = 0.0198).

Supplementary Figure 3 After filtering the taxa, a differential abundance analysis was performed on the remaining taxa. **(A,B)** *n* = 24 significantly differential clusters were observed in AB, WH, and HC. **(C,D)** Further subclassifying the group into atopic and non-atopic level, *n* = 42 clusters could be calculated in atopic AB and WH (*n* = 50).

Supplementary Figure 4 The effect of *Staphylococcus aureus* and *Haemophilus influenzae* on nasal epithelial spheroid cultures. After 24 h of infection, a significant release of the IL-33, TNF-α, and IL-17A concentrations in the supernatants was observed particularly in exposure experiments with *S. aureus* and for IL-33 in *H. influenzae* exposure experiments. ^#^The non-provoked cells showed values under the detection and the measurable reference values were used for analyses.

Supplementary Figure 5 The beta diversity of diseased patients showed differential variances between atopy vs. non-atopy subjects. **(A,B)** For the β-diversity, we analyzed different variants of between-sample diversity, Jaccard for presence/absence-based with no phylogenetic information, Bray-Curtis for abundance-based with no phylogenetic information, Unifrac for presence/absence-based with phylogenetic information and wUnifrac for abundance-based with phylogenetic information. A permdisp test, showed an interesting homogeneity of WH in comparison to AB or HC subjects. In addition, using 2D space Multi-Dimensional Scaling, WH were highly homogeneous (AB vs. WH *p* = 0.00089; WH vs. HC *p* = 0.031; AB vs. HC *p* = 0.768). (AB, Asthmatics; WH, Wheezers; HC, Healthy controls).

Supplementary Table 1 Differential abundant taxa and description of significant clusters of the AB, the WH, and the HC.

Supplementary Table 2 Differential abundant taxa and description of significant clusters of the atopic AB, and the WH, in comparison to the HC.

## Abbreviations

AB: Asthmatics
DRKS: Deutsches Register für Klinische Studien (German Clinical Trials Register)
HC: Healthy controls
IL: Interleukin
LDH: Lactate dehydrogenase
MALDI-TOF-MS: Matrix-assisted laser desorption/ionization time-of-flight mass spectrometry
MDS: Multi-Dimensional Scaling
NAEsp: Human nasal epithelial spheroids
NGS: Next-generation sequencing
NP: Nasopharynx
WHO: World Health Organization
OP: Oropharynx
OTU: Operational taxonomic unit
WH: Wheezers.
