# Supplementary figures and images for: The Rhinobiome of Exacerbated Wheezers and Asthmatics: Insights From a German Pediatric Exacerbation Network
Source: Front Allergy. 2021 May 31;2:667562. doi: 10.3389/falgy.2021.667562 (PMC8974812; doi:10.3389/falgy.2021.667562)

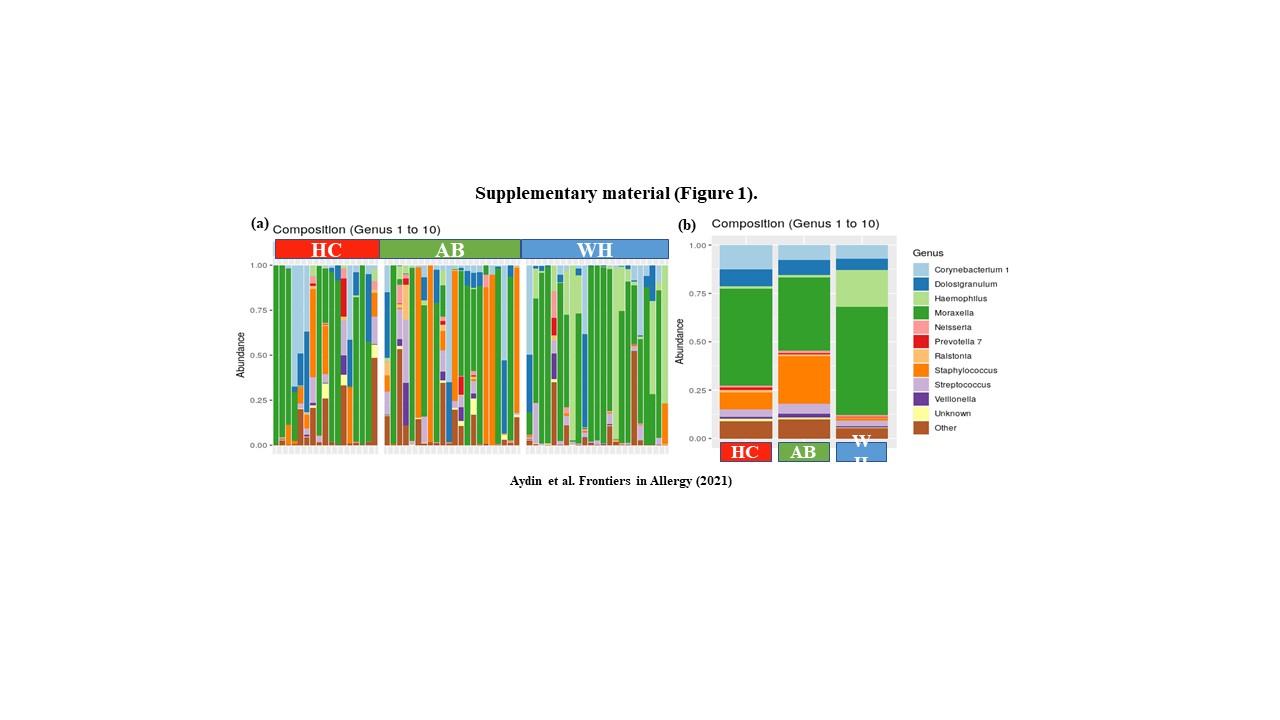

Supplement: Supplementary Figure 1 — (A,B) The data presents the genus level taxonomy. [file Image_1.JPEG]

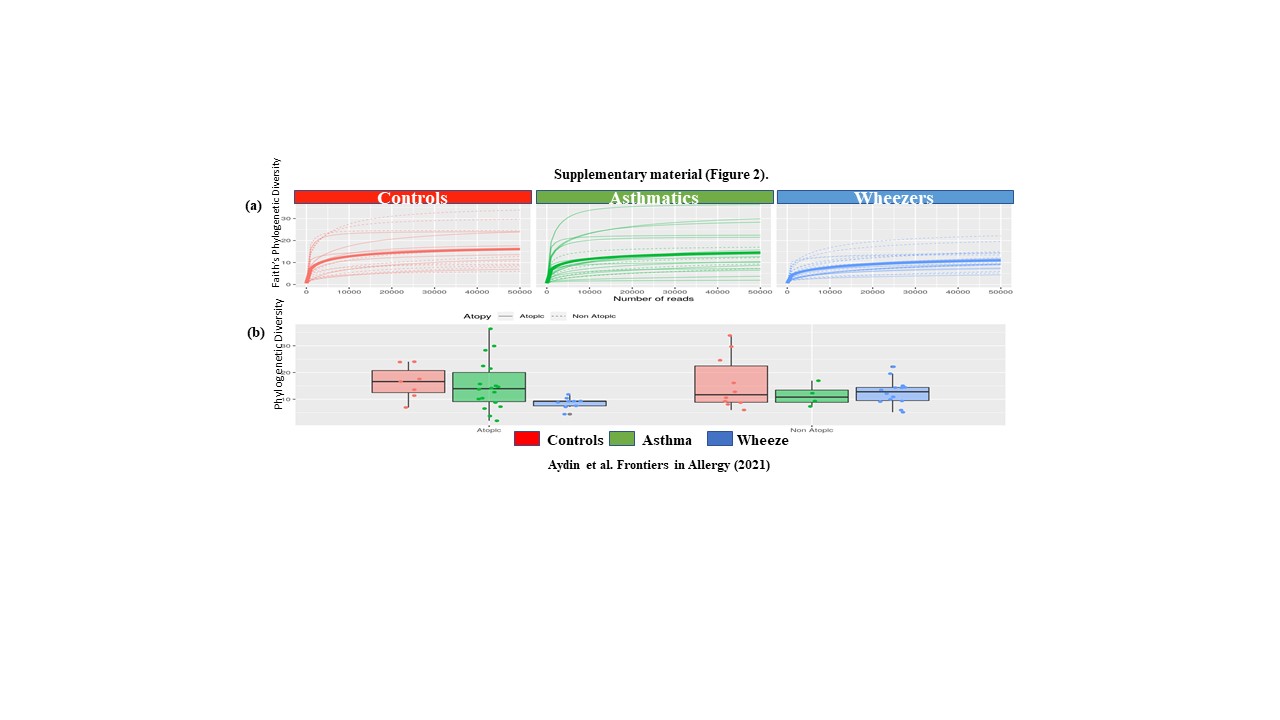

Supplement: Supplementary Figure 2 — The phylogenetic diversity. (A) The phylogenetic diversity contrasting pair with reads in HC, AB, and WH. (B) The atopic WH were significantly different from the atopic AB (p = 0.05), as well as the atopic vs. the non-atopic WH (p = 0.0198), and the atopic vs. non-atopic AB (p = 0.0198). [file Image_2.JPEG]

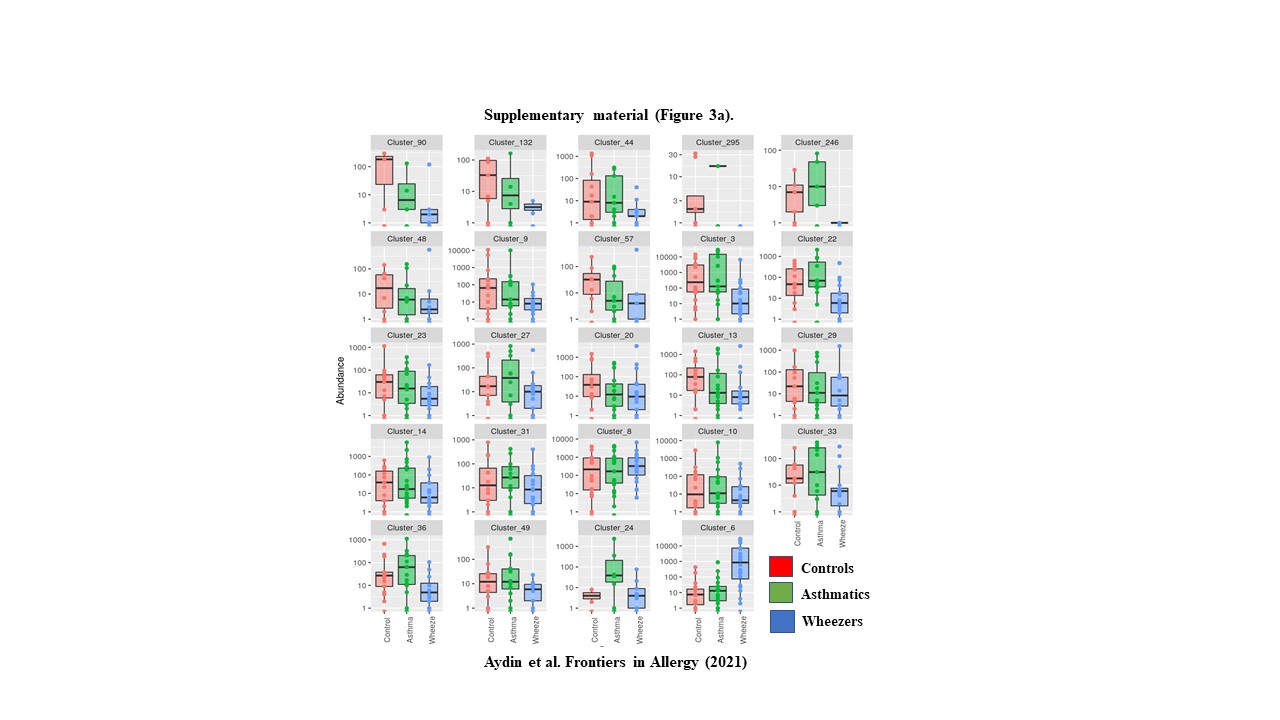

Supplement: Supplementary Figure 3 — After filtering the taxa, a differential abundance analysis was performed on the remaining taxa. (A,B) n = 24 significantly differential clusters were observed in AB, WH, and HC. (C,D) Further subclassifying the group into atopic and non-atopic level, n = 42 clusters could be calculated in atopic AB and WH (n = 50). [file Image_3.JPEG]

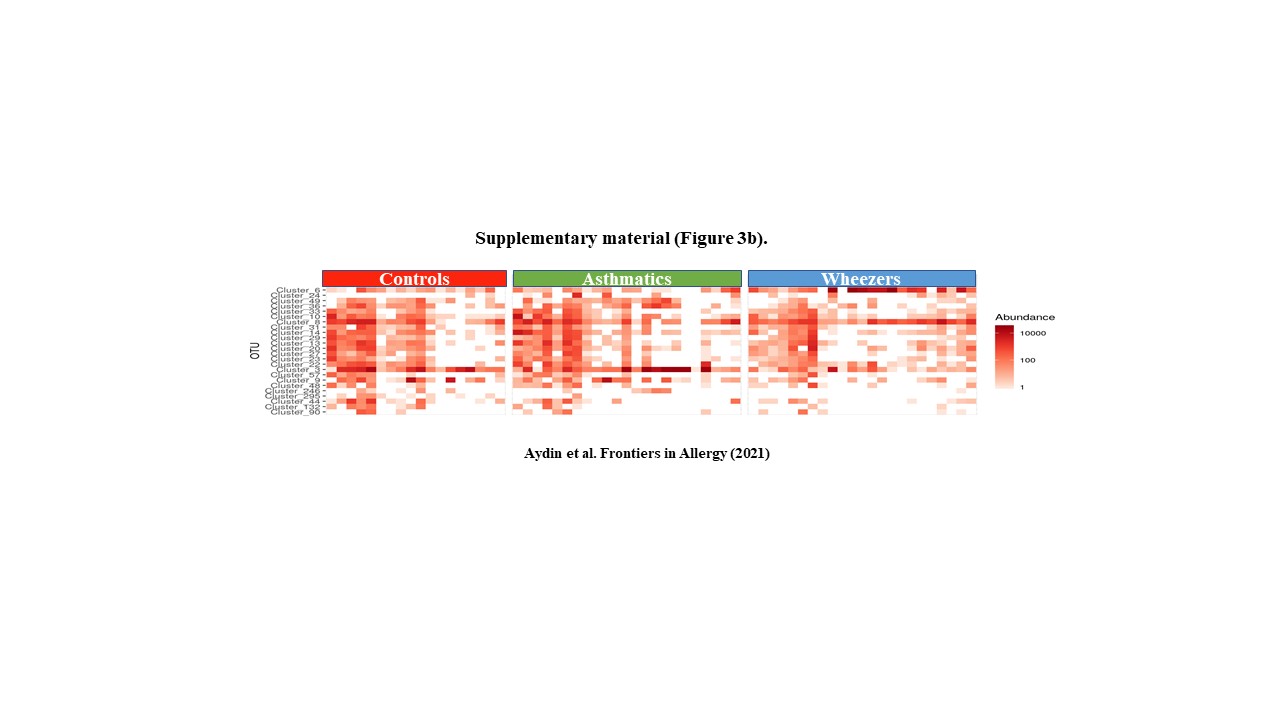

Supplement: Supplementary file 4 [file Image_4.JPEG]

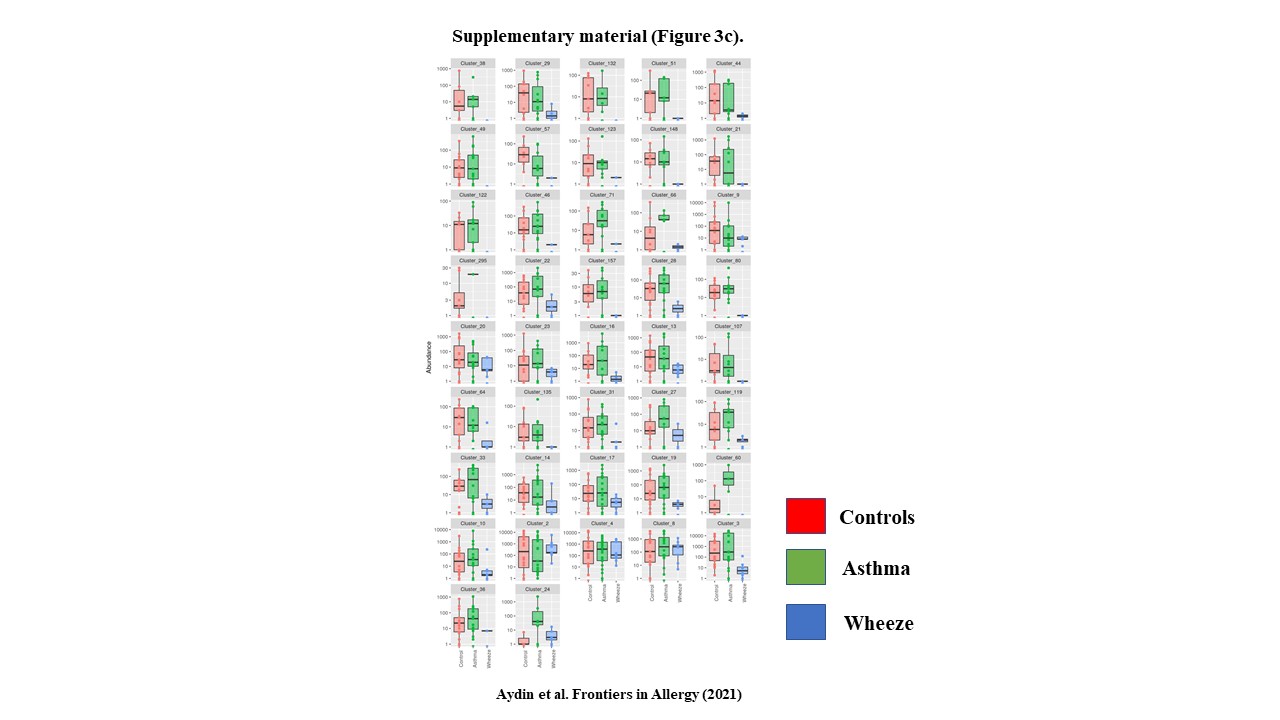

Supplement: Supplementary file 5 [file Image_5.JPEG]

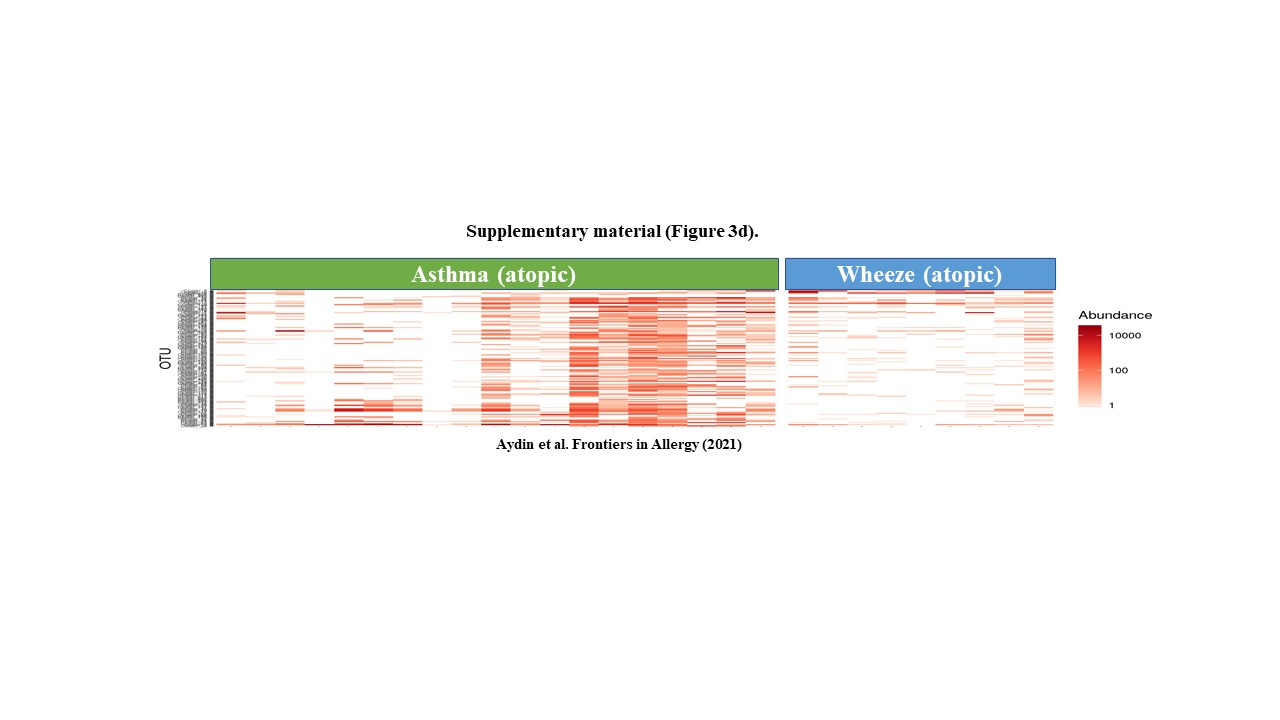

Supplement: Supplementary file 6 [file Image_6.JPEG]

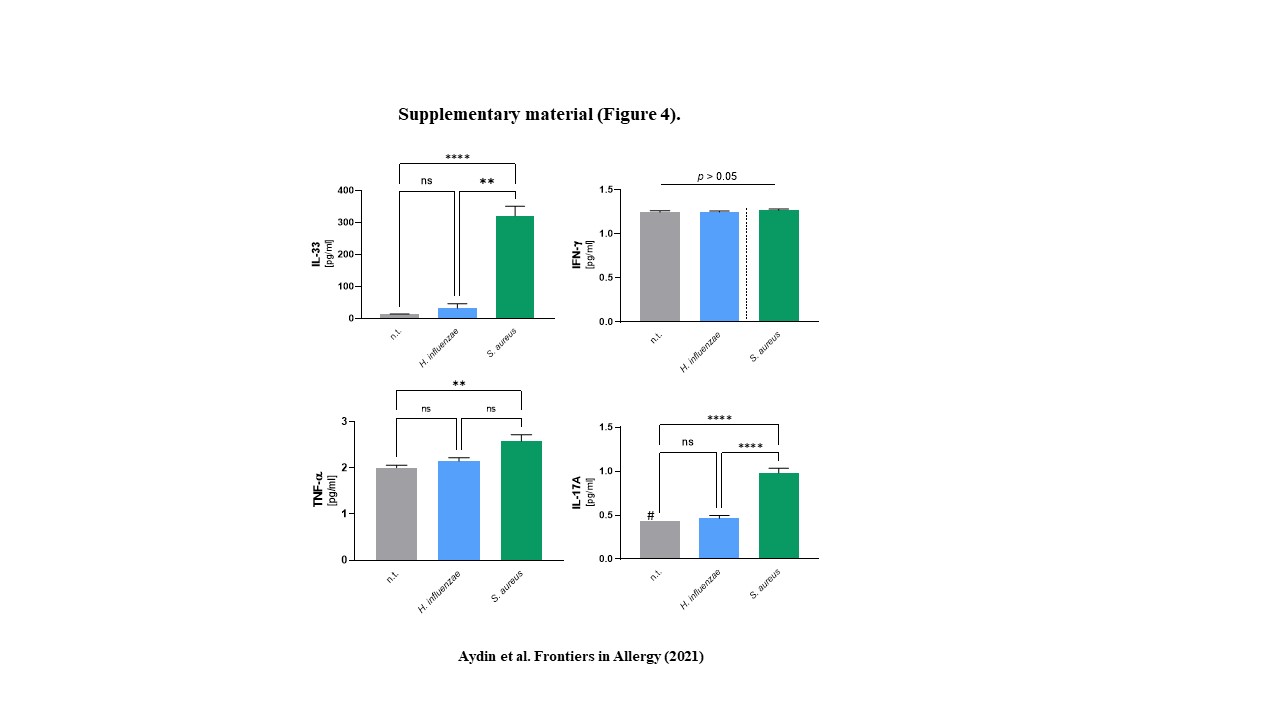

Supplement: Supplementary Figure 4 — The effect of Staphylococcus aureus and Haemophilus influenzae on nasal epithelial spheroid cultures. After 24 h of infection, a significant release of the IL-33, TNF-α, and IL-17A concentrations in the supernatants was observed particularly in exposure experiments with S. aureus and for IL-33 in H. influenzae exposure experiments. #The non-provoked cells showed values under the detection and the measurable reference values were used for analyses. [file Image_7.JPEG]

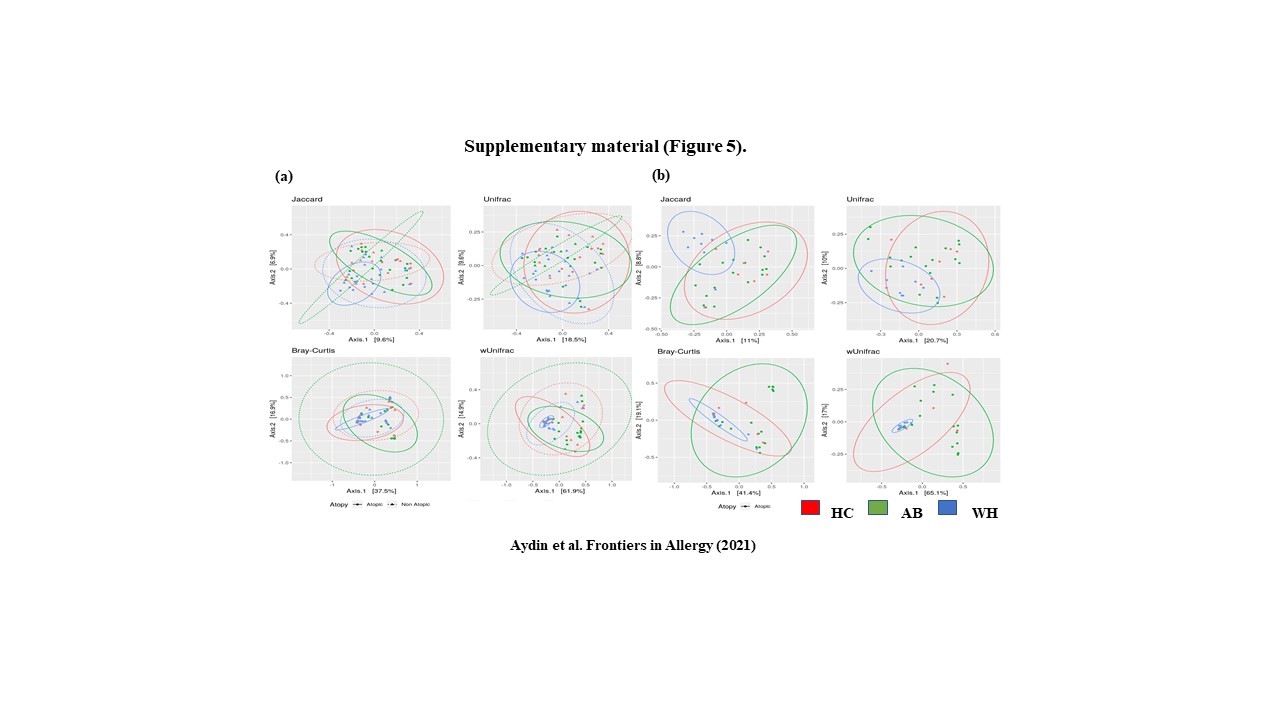

Supplement: Supplementary Figure 5 — The beta diversity of diseased patients showed differential variances between atopy vs. non-atopy subjects. (A,B) For the β-diversity, we analyzed different variants of between-sample diversity, Jaccard for presence/absence-based with no phylogenetic information, Bray-Curtis for abundance-based with no phylogenetic information, Unifrac for presence/absence-based with phylogenetic information and wUnifrac for abundance-based with phylogenetic information. A permdisp test, showed an interesting homogeneity of WH in comparison to AB or HC subjects. In addition, using 2D space Multi-Dimensional Scaling, WH were highly homogeneous (AB vs. WH p = 0.00089; WH vs. HC p = 0.031; AB vs. HC p = 0.768). (AB, Asthmatics; WH, Wheezers; HC, Healthy controls). [file Image_8.JPEG]
