# Supplementary material for: The Rhinobiome of Exacerbated Wheezers and Asthmatics: Insights From a German Pediatric Exacerbation Network
Source: Front Allergy. 2021 May 31;2:667562. doi: 10.3389/falgy.2021.667562 (PMC8974812; doi:10.3389/falgy.2021.667562)
Supplement: Supplementary Table 1 — Differential abundant taxa and description of significant clusters of the AB, the WH, and the HC. [file Data_Sheet_2.docx]

Supplementary Table 1. Differential abundant taxa and description of significant clusters of the AB, the WH and the HC (n = 24 clusters).

Supplementary Table 2. Differential abundant taxa and description of significant clusters of the atopic AB, and the WH, in comparison to the HC (n = 42 clusters).
