# Supplementary material for: The Rhinobiome of Exacerbated Wheezers and Asthmatics: Insights From a German Pediatric Exacerbation Network
Source: Front Allergy. 2021 May 31;2:667562. doi: 10.3389/falgy.2021.667562 (PMC8974812; doi:10.3389/falgy.2021.667562)
Supplement: Supplementary file 10 [file Data_Sheet_1.DOCX]

Supplementary material

1. Technical information

*1.1. DNA isolation of nasal swabs*

The DNA isolation was performed with the QIAamp DNA Micro Kit and the Pathogen Lysis Tubes of the QIAamp DNA Microbiome Kit were separately used for mechanical lysis. The technical steps for sample preparation were taken from QIAamp DNA Mini and Blood Mini Handbook, Appendix G: Protocol for Eye, Nasal, or Pharyngeal Swabs. The further steps were based on the QIAamp DNA Micro Handbook, Protocol: Isolation of Genomic DNA from Small Volumes of Blood" and were individually adapted under recommendation of QIAamp.

Briefly, the protocol was established as follows: 1) At first, the swab tubes were systematically thawed. 2) The tip was transferred to a 15 ml Falcon tube and 500 µl PBS was added. 3) The swab was shaken, were transferred to a 1,5 ml sample tube, and centrifuged at 5,000 x g for 10 min. 4) The supernatant was carefully removed, and the pellet was resuspended in 400 µl buffer ATL incl. reagent DX. 5) The sample was transferred to the Pathogen Lysis tubes. The mechanical lysis was performed at a TissueLyser LT for 10 min at 50 Hz or at a TissueLyser II for 10 min at 30 Hz (or on a vortex with foam attachment for 10 min at max. speed). 6) The Pathogen Lysis Tubes were centrifuged at 10,000 x g for 1 min. The liquid was carefully mixed and transferred into a fresh sample tube. 7) 20 µl Proteinase K was then added, vortexed for 15 sec and incubated for 1 hour at 56°C on a shaker at 900 rpm. 8) 400 µl Buffer AL was pipetted, vortexed for 15 sec and incubated for 10 min at 70°C on a shaker at 900 rpm. 9) The samples were washed with 200 µl ethanol 100%, vortexed for 15 sec and incubated for 3 min at room temperature. 10) After centrifugation, 700µl of the contents was transferred into a Qiamp MinElute colum and centrifuged at 6,000 x g for 1 min. The rest of the lysate from the sample tube was transferred into the MinElute Spin Column and centrifuge again at 6,000 x g for 1 min. 11) The MinElute column was transferred into a clean 2ml collection tube, 500 µl Buffer AW1 was added and centrifuged at 6,000 g for 1 min. 12) The MinElute column was transferred into a clean 2 ml collection tube and 700 µl Buffer AW2 was added followed by a centrifugation step at 6,000 g for 1 min. 13) The MinElute column was again transferred into a clean 2 ml collection tube, 700 µl EtOH (96-100%) was added to the membrane and centrifuged 1 min at 6000 x g. 14) The MinElute column was transferred into a clean 1.5 ml microcentrifuge tube, the MinElute column was opened and incubated at room temperature for 10 min. 15) 20-100 µl AE/ ATE/distilled water (pH ≥ 7.0) was directly pipetted to the center of the membrane and incubated for 5 min at room temperature. After the final centrifugation step at 20,000 x g for 1 min, the samples were deep-freezed at -20°C until library preparation.

*1.2. 16S rRNA gene amplicon sequencing library preparation protocol*

The 8,939,302 sequences are grouped in 693,986 OTUs - the biggest OTU contains 3,313,690 sequences – 672,748 OTUs (96.9% of sequences) are formed from one sequence (called singleton) – 10 OTUs contain 25% of sequences. Chimera is always a big proportion of sequences obtained by sequencing 16S amplicons with Illumina® technology, mainly due to successive PCR. This tool uses vsearch described by Rognes *et al.* (2016) in a de novo mode to detect them after a cross-samples validation. The chimera report shows that 3.5% of OTUs are detected as chimeric and removed. They represent 0.9% of all sequences. Via the FROGS filters we removed singletons which are mainly chimeric or noised sequences. The first filter process allowed to keep 8,205,207 sequences (92.6%) if we remove only the singletons (97.9% of all OTUs). 14,198 OTUs are remaining. To remove other low-abundant OTUs, we can remove OTUs viewed less than 10 times overall samples. The final filters report shows that we keep 8,171,007 sequences (92.2%) if we filter at 10 sequences. 1,847 OTUs are remaining. The affiliation report and affiliation stats report show that 99.9% of sequences are affiliated (only 7,271 sequences not affiliated). 90.46% of sequences are multi-affiliated at Species rank (expected for 16S amplicons) and 0.6% are multi-affiliated at Genus rank. A small fraction of sequences has a hit against the database with less than 95% of identity and less than 95% of coverage. At least 29,578 reads are available for each sample, and at least 50,000 for all but 2 samples. All curves demonstrate a plateau that is reached quite quickly (around 30,00 reads the leftmost vertical bar). If we accept to lose the two lowly sequenced samples, we can keep 50,000 reads per sample (rightmost vertical bar) and get closer to the plateau for most curves.

1.3. Proteomics

*1.3.1. Sample Preparation for MS/MS Analysis*

Samples were diluted with lysis buffer to a final concentration of 50mM triethylammonium bicarbonate, 1% (w/v) sodium deoxycholate, 4% (w/v) sodium dodecyl sulphate, and 100 mM dithiothreitol, incubated for 8 min at 95 °C and sonicated for 5 min. Subsequently glycerin was added to a final concentration of 20% (v/v) and samples were separated by 1D SDS PAGE providing 1 cm separating distance. Proteins were in gel digested and prepared for MS/MS analyses as previously described (Bonn, F, Bartel, J, Büttner, K, Hecker, M, Otto, A and Becher, D (2014)). Picking vanished proteins from the void: how to collect and ship/share extremely dilute proteins in a reproducible and highly efficient manner. Anal. Chem. 86, 7421–7427). Briefly, gel lanes were cut resulting in two gel pieces per sample, gel pieces were cut into smaller blocks and transferred into low binding tubes. Samples were destained and dried in a vacuum centrifuge before being covered with trypsin solution. Digestion was carried out at 37 °C overnight before peptides were eluted in water by ultrasonication. The peptide-containing supernatant was transferred into a fresh tube, desiccated in a vacuum centrifuge and peptides were resolubilized in 0.1% (v/v) acetic acid for mass spectrometric analysis.

*1.3.2. MS/MS Analysis*

LC-MS/MS analyses were performed on an LTQ Orbitrap Velos Pro (ThermoFisher Scientific, Waltham, Massachusetts, USA) using an EASY-nLC II liquid chromatography system. Tryptic peptides were subjected to liquid chromatography (LC) separation and electrospray ionization-based mass spectrometry (MS) applying exactly the same injected volumes in order to allow for label-free relative protein quantification. Therefore, peptides were loaded on a self-packed analytical column (OD 360 μm, ID 100 μm, length 20 cm) filled with 3 µm diameter C18 particles (Dr. Maisch, Ammerbuch-Entringen, Germany) and eluted by a binary nonlinear gradient of 5 - 99 % acetonitrile in 0.1 % acetic acid over 151 min with a flow rate of 300 nL/min. For MS analysis, a full scan in the Orbitrap with a resolution of 30,000 was followed by collision-induced dissociation (CID) of the twenty most abundant precursor ions. MS2 experiments were acquired in the linear ion trap.

*1.3.3. MS Data Analysis*

Database search was performed with a database containing Uniprot-entries of *M. catarrhalis* (former *Branhamella catarrhalis*), protein sequences of M. catarrhalis strains BBH18, BC8, O35E, *Moraxella sp.* HMSC061H09, and various known *Moraxella* phages (Mcat) (downloaded on 5th November 2019, 15694 entries) and a human protein database downloaded from Uniprot at 15th July 2019 (20428 entries) as well as common laboratory contamintation and a reverse entry for every forward entry (72330 entries in total). Sorcerer-SEQUEST 4 (Sage-N Research, Milpitas, USA) was applied for database searching with activated TPP options, fully specific tryptic cleavage (KR/P) with up to two missed cleavages and methionine oxidation (+15.99 Da) as variable modification. Precursor mass tolerance was set to 10 ppm and fragment mass tolerance was set to 1.0005 Da. The Trans Proteomic Pipeline (TPP, 5.1.0-rc1 Sysygy, 2017) was applied for further processing of the mzXML and pep.XML files from the Sorcerer output. Spectral library creation was performed according to Schubert et al. (Schubert, Olga T.; Gillet, Ludovic C.; Collins, Ben C.; Navarro, Pedro; Rosenberger, George; Wolski, Witold E. et al. (2015): Building high-quality assay libraries for targeted analysis of SWATH MS data. In: Nature protocols 10 (3), S. 426–441. DOI: 10.1038/nprot.2015.015.) with slight modifications. Briefly, spectra obtained during analysis of a pure culture of five *Moraxella* strains and their particular identifications were linked together and entries from all datasets were combined to interact.pep.XML files (InteractParser). The following settings were used: minimum peptide sequence length of seven amino acids, use accurate mass binning, using: ppm, ignore charge states: 1+ and higher than 4+. Then all identified spectra with a peptide probability allowing for an FDR of 0.01 at protein level (iProphet and Mayu algorithm) were imported to build a spectral library using SpectraST. Decoy consensus spectra were generated in the TPP and appended to the consensus spectra of positively identified peptides.

2. Additional results

*2.1. Exacerbated AB exhibit more abundance variability than WH and HC, whereas WH are homogeneously abundant.*

For the β - diversity, we analyzed different variants of between-sample diversity, Jaccard for presence/absence-based with no phylogenetic information, Bray-Curtis for abundance-based with no phylogenetic information, Unifrac for presence/absence-based with phylogenetic information and wUnifrac for abundance-based with phylogenetic information. For the β - diversity, we analyzed different variants of between-sample diversity, Jaccard for presence/absence-based with no phylogenetic information, Bray-Curtis for abundance-based with no phylogenetic information, Unifrac for presence/absence-based with phylogenetic information and wUnifrac for abundance-based with phylogenetic information. A PERMANOVA test showed modest differences between WH, AB and HC subjects (p = 0.013, R2 = 12%). The groups differed in other aspects: WH subjects add homogeneous communities whereas both AB and HC subjects add much more diverse taxonomic profiles (WH vs AB, p = 0.0075; WH vs. HC p = 0.047; AB vs. HC p = 0.88, permdisp test for homogeneity of dispersion) *(Supplementary Figure 5a+b).*
